# Colony size measurement of the yeast gene deletion strains for functional genomics

**DOI:** 10.1186/1471-2105-8-117

**Published:** 2007-04-04

**Authors:** Negar Memarian, Matthew Jessulat, Javad Alirezaie, Nadereh Mir-Rashed, Jianhua Xu, Mehri Zareie, Myron Smith, Ashkan Golshani

**Affiliations:** 1 Department of Electrical and Computer Engineering, University of Toronto, Toronto, Canada; 2 Department of Electrical Engineering, Ryerson University, Toronto, Canada; 3 Department of Biology, Carleton University, Ottawa, Canada; 4 Department of Systems Design Engineering, University of Waterloo, Waterloo, Canada; 5 The Best Institute, University of Toronto, Toronto, Canada; 6 Department of Molecular and Medical Genetics, University of Toronto, Toronto, Canada; 7 Ottawa Institute of System Biology, Ottawa, Canada

## Abstract

**Background:**

Numerous functional genomics approaches have been developed to study the model organism yeast, *Saccharomyces cerevisiae*, with the aim of systematically understanding the biology of the cell. Some of these techniques are based on yeast growth differences under different conditions, such as those generated by gene mutations, chemicals or both. Manual inspection of the yeast colonies that are grown under different conditions is often used as a method to detect such growth differences.

**Results:**

Here, we developed a computerized image analysis system called Growth Detector (GD), to automatically acquire quantitative and comparative information for yeast colony growth. GD offers great convenience and accuracy over the currently used manual growth measurement method. It distinguishes true yeast colonies in a digital image and provides an accurate coordinate oriented map of the colony areas. Some post-processing calculations are also conducted. Using GD, we successfully detected a genetic linkage between the molecular activity of the plant-derived antifungal compound berberine and gene expression components, among other cellular processes. A novel association for the yeast *mek1 *gene with DNA damage repair was also identified by GD and confirmed by a plasmid repair assay. The results demonstrate the usefulness of GD for yeast functional genomics research.

**Conclusion:**

GD offers significant improvement over the manual inspection method to detect relative yeast colony size differences. The speed and accuracy associated with GD makes it an ideal choice for large-scale functional genomics investigations.

## Background

The increasing number of completed genome sequencing projects has provided biologists with a wealth of sequences containing thousands of genes. Many of these genes code for proteins of unknown function. This has led to the development of several large-scale methodologies that are grouped together under the term functional genomics/proteomics, with the aim of investigating the putative gene functions [1-3]. In the context of functional genomics, yeast *Saccharomyces cerevisiae *has emerged as the model organism of choice for studying eukaryotic cells. *S. cerevisiae *has been the target of numerous large-scale experiments including genome sequencing [4], expression profile [5], interaction mapping [6], etc. While much has been learnt in the recent years, more experiments are needed to uncover the details of the cell's behavior and responses to internal and external stimuli at the molecular level.

One way of defining protein functions is via the interactions that the proteins make with each other. It is generally accepted that the proteins that are functionally related, often interact with each other and form functional protein complexes [7]. Consequently, protein-protein interaction (PPI) analysis has been used as a method to assign novel functions to proteins in model organisms such as *S. cerevisiae *and *Escherichia coli *[8,9].

Genetic interaction analysis is another method which is used to study gene function. It is based on the assumption that many genes and pathways in yeast and other eukaryotic cells may be functionally redundant so that in the absence of a target gene, there will be other genes that compensate for its loss of activity with no phenotypic consequences [10]. However, the deletion of a second functionally related gene may result in sickness or lethality. Therefore, the sickness of double mutants or "synthetic lethality" identifies genetic and hence functional relationships between the two genes [11,12]. The sickness or lethality in this case is determined by colony size reduction.

The availability of genome sequencing data has also greatly impacted studying the genetics of disease. Chemical genetics, which is positioned at the borders of chemistry and genetics, has received considerable attention because it may hold a key to the discovery of drugs that affect the molecular mechanism of different diseases. Using the set of non-essential yeast gene deletion (approximately 4500) strains, very recently it was demonstrated that chemical-genetic analysis provided a fast and efficient way to determine the targets of inhibitory compounds [13]. This was done by analyzing the growth of the yeast gene deletion strain colonies in the presence of the sub-lethal concentration of a bioactive compound. Analyses of the gene mutations that cause hypersensitivity to the compounds were then used to identify the cellular targets of the bioactive compounds.

Both commercial and free image analysis tools have previously been developed for analyzing biological images. Some of these tools are designed for specific functions such as cell or colony counting [14,15], whereas others are more dynamic and permit some user interactive analysis of individual images [16].

Here, we report the development of a computerized system termed Growth Detector (GD) that performs accurate yeast colony area measurement and calculates required statistical parameters which is suitable for comparative analysis of two yeast colony arrays. We use this automated tool to study the molecular mechanism by which berberine affects eukaryotic cells. In addition, GD analysis reveals a novel function for the *mek1 *gene in DNA repair pathway, further indicating the use of GD in biological investigations.

## Results and Discussion

### The efficiency of GD to detect growth differences

We evaluated the ability of GD to detect growth differences in yeast colonies, by comparing colony growth for yeasts treated with various compounds to the untreated ones (used as control). For the description of how the GD analysis works please refer to the Methods section. In our experiment with cobalt, we pinned approximately equal number of mutant yeast cells, for 384 strains, on media containing either a sub-lethal concentration of cobalt (400 ug/ml), or no additional cobalt. The GD analysis and the manual (visual) inspection (MI) method were used to evaluate the colony growth differences. The results were used to evaluate the efficiency of GD to identify colony size differences.

The MI method was performed by looking at the images of the two plates, by eye, and estimating the relative growth of a target colony over the average growth on the same plate and comparing it to that on the control plate. It should be noted that the treatment of the cells often results in an overall growth reduction for the entire array of yeast colonies, which to some extent may complicate manual comparisons. As indicated in Table 1, the MI approach resulted in the detection of 15 strains as positives (sensitive to 400 ug/ml of cobalt). Aside from the subjective nature of this approach which could complicate the intrepretation of the results, the MI analysis is also laborious. On average, it takes approximately 5 seconds for each yeast colony to be accurately analyzed.

**Table 1:** Comparison of sensitivity detection for yeast strains using different approaches

Method	Total sensitives detected	Sensitives shared with MI	% of MI covered^a^	Sensitives shared with GD	% of GD covered^b^	Sensitives shared with ST	% of ST covered^c^	Novel/false positives^d^	% of novel/false positives^e^
Manual Inspection (MI)	15	N/A	N/A	11	38%	6	32%	9	60%
Growth Detector (GD)	29	11	73%	N/A	N/A	12	63%	17	59%
Spot Test (ST)	19	6	40%	12	41%	N/A	N/A	N/A	N/A

Cell sensitivity to cobalt (400 ug/ml) for 384 different yeast strains was analyzed using colony size reduction (MI and GD) or number of colonies formed (ST). In addition to the 15 strains detected by MI, GD identifies another 14 sensitive strains. The data gathered by GD has more overlap (63%) with the ST data as compared to those gathered by MI (32%). Assuming that the ST data represent ground truth, MI and GD have comparable % of novel/false positives which is calculated using 100 × (Novel/false positives)/(Total sensitives detected). ^a,b ^and ^c ^are calculated using 100 × (Sensitives shared with MI)/(Total sensitives detected by MI), 100 × (Sensitives shared with GD)/(Total sensitives detected by GD), and 100 × (Sensitives shared with ST)/(Total sensitives detected by ST), respectively. ^d ^and ^e ^assume that the ST data represent ground truth. N/A is not applicable.

The performance time of the MI method is compared to approximately 30 seconds detection and statistical calculation time for all 384 yeast strains using the GD analysis. This is approximately a 64-fold increase in performance time. Additionally, GD detected 29 sensitive strains (Table 1). This is compared to the 15 strains identified by the MI analysis and suggests a 2-fold increase in detection of target strains for GD versus MI. Consequently, for a complete experiment (approximately 4500 strains), more than 160 target sites might be identified with the GD analysis that could be missed otherwise. This is a very significant improvement for biological investigations especially given the accuracy of GD (see the discussion below).

In addition to the improved speed and effectiveness of GD over MI, there are other advantages to using this automated method over the manual approach. GD can provide quantitative information about the observed growth differences. This quantitiative information can be used to prioritize the target candidates for follow-up experiments. Multiple positive hits often present a challenge for deciding the priority of each hit. Once the hits are quantified however, those with the best scores get automatic priority. In addition, GD analysis may have the advantage of reducing the amount of starting material needed for cell sensitivity screens. Due to the constraints associated with manual scoring, each experiment may need to be repeated several times. Since the pure amount of the experimental bioactive chemicals is often a limiting factor [17], large-scale experiments with certain valuable samples for which only small quantities were readily available, were previously thought to lack merit. GD analysis can change this.

**Figure 1:**
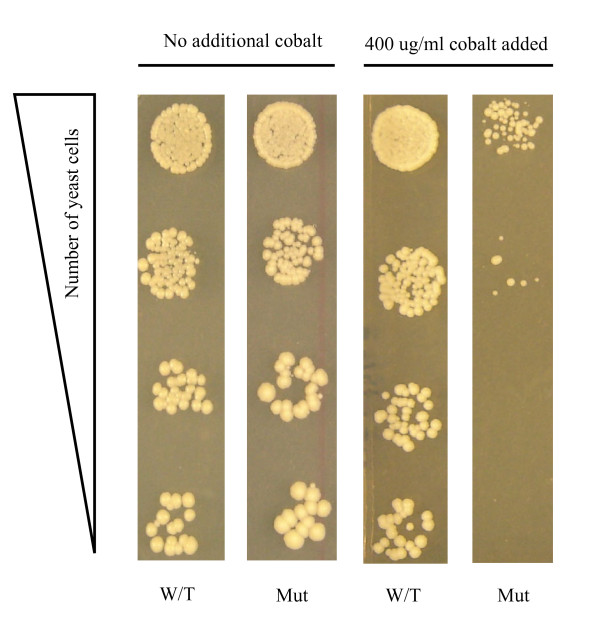
**Spot Test analysis**. Equal numbers of wild type or mutant yeast cells were spotted on media with or without 400 ug/ml of cobalt. The relative numbers of yeast cells spotted on the plates are indicated. In relation to wild type, the mutant strain did not produce as many colonies in the presence of cobalt. This suggests sensitivity to cobalt for the mutant cells. W/T is the wild type (normal) strain; Mut is the gene deletion mutant strain for the *rox1 *gene.

We also used spot test (ST) analysis as an alternative method for detecting the sensitivity of yeast strains to cobalt (see Figure 1). It should be noted however, that the determination of cell sensitivity to a bioactive compound is a subjective matter [18]. A cell in a specific experimental condition may show certain sensitivity to a compound. However, small variations in the experimental conditions (for example, small changes in growth temperature, liquid versus solid media, etc) may reduce or enhance this sensitivity. Therefore, while there is a positive correlation between sensitivities detected by spot test and colony size reduction analyses [11], the results are not expected to fully overlap. For practical reasons however, here we used the ST analysis as an alternative method for detecting sensitivity to which colony size reduction analysis may be compared.

To do the ST analysis, wild type (normal) yeast cells as well as the mutants were grown in liquid medium to saturation. The cells were diluted 50 times and allowed to grow to mid-saturation (mid-log). The cells were then diluted using serial-dilution and decreasing numbers of cells were plated on the solid media with or without (used as a control) the target bioactive compound. The relative reduced number of colonies formed is scored as sensitivity to the compound. An example of this is shown in Figure 1. The first circle on the top for each experiment contains 10^4 ^cells. The following spots contain approximately 10^3^, 10^2 ^and 10^1 ^cells, respectively. This procedure is very time consuming and is done over a course of 4 days. In our experiments, we analyzed 16 strains per day. The analysis of the 384 strains by this technique took 28 full days (this includes some repeat). Consequently, while the results obtained are reliable, this technique can hardly replace the colony size reduction approach for large-scale analysis.

As indicated in Table 1, the ST analysis identified 19 sensitive strains (positives). Of these 19 strains, 6 and 12 strains were also identified as positives by the MI and GD analyses, respectively, indicating an overlap of 32% and 63% for the data gathered by MI and GD with that of ST, respectively. This provides further evidence for the suitability of the GD analysis to identify sensitive strains and reaffirms its advantage over the MI analysis. Further, MI and GD identified 9 and 17 strains as positives, respectively, which did not appear as sensitives using the ST analysis. These may represent novel positives, and may stem from the differences in the cell sensitivities under different experimental conditions. Alternatively, they may represent false-positives if the strains identified by ST are assumed as the true sensitives.

We then examined if GD can potentially identify all positives detected by MI and ST if a high rate of false positives was tolerated. It was observed that all the sensitives detected by MI can also be detected by GD if the selection threshold for defining sensitivity in GD analysis is lowered from 30% colony growth difference to 19%. This lowering of the threshold also resulted in the detection of 49 false/novel positives. The lowering of the stringency for the sensitivity selection in GD analysis from 30% growth reduction to 23% resulted in the detection of 3 additional sensitives that were originally detected by ST alone, as well as 27 false/novel positives. Further lowering of this threshold however, did not result in the detection of the remaining 4 sensitives (of a total of 19) that were detected by ST alone. The fact that GD cannot detect all the sensitives detected by ST further highlights the difference between the data collected by the two approaches. Also this difference might be partially explained by the experimental errors associated with the two methods.

Altogether, results indicate that the automated system (GD) offers significant improvement over the manual scoring (MI) of yeast colonies. Sensitive additional information can now be extracted in a timely fashion from the same experiment, which otherwise would have been missed. This additional information can help biologists to better interpret their results.

During the preparation of this manuscript a new image analyzer termed CellProfiler was developed which besides its use in analyzing various cell and colony images, it is also suitable for growth analysis of yeast colony arrays [19]. Like GD, CellProfiler is also developed in MATLAB and measures the amount of growth in each patch of yeast, using intensity units and shape-related measures. The main advantage of GD over CellProfiler is that in addition to providing an accurate coordinate oriented map of colony areas, GD also performs post processing calculations and attains useful statistical information about the growth pattern of yeast colonies within each plate and relative to other plates (comparative analysis). Another advantage of GD over CellProfiler is its ability to compensate for plates lacking entire edge rows or columns. For the purpose of functional genomics and in light of the growing number of cell colony arrays [20,21], the need for automated tools, such as GD, which are capable of performing comparative image analysis is apparent.

### Molecular mechanism of berberine

The alkaloid berberine is a component of a number of important medicinal plants that are used in traditional Chinese and Ayurvedic medicine [22]. Berberine has demonstrated significant antimicrobial activity against different organisms including fungi [23,24] and is relatively nontoxic to humans [25]. This makes berberine an ideal candidate for pharmaceutical drug development from medicinal plants. However, little information is available about the molecular mechanism by which this bioactive compound affects the cell. To investigate the mechanism of the bioactivity of berberine, we used GD analysis to examine the growth of the yeast non-essential gene deletion array (approximately 4500) in the presence and the absence of the sub-inhibitory concentration (70 μg/ml) of berberine. The most sensitive yeast deletion strains (195) detected by GD were used for further analysis.

We then randomly selected 10 of these strains and examined their hypersensitivity to berberine by minimum inhibitory concentration (MIC) assay. This was carried out by placing approximately 1000 yeast cells in each well of a 96-well microtiter plate in the presence of a gradient of berberine concentration varying from 6.7 to 1 × 10^-5 ^mg/ml and comparing their growth to that of the wild type strain for each of the 96 varying concentrations of berberine. As expected, all 10 examined deletion strains had significantly lower MIC values as compared to the wild type strain, further reaffirming that GD analysis can correctly identify drug sensitive strains.

Of the sensitive strains, only 140 had deletion of the genes with known functions. 33 of those genes were associated with stress response or multi-drug resistance. These genes are thought to be involved in general stress conditions and therefore may not be specific to the molecular activity of berberine. Consequently, they were eliminated from further analysis to study the molecular target of berberine. Analysis of the functions of the remaining 107 genes indicated that 41 of them had a role in the process of gene expression. This suggests that berberine is genetically linked to gene expression components, among other cellular processes. The smaller identified gene functional clusters had roles in the proccesses such as metabolism (21 genes) and cell wall synthesis (7 genes). It should be noted that these observations are considered preliminary and further investigations are needed to elucidate the exact mechanism of berberine activity.

### Identification of a novel role for *mek1 *in DNA damage repair

To identify novel genes involved in DNA damage repair (DDR) we examined the set of yeast non-essential gene deletion strains for their hypersensitivity to methyl methanesulfonate (MMS), a known DNA damaging agent, by the pinning approach. GD analysis was then used as before to compare the colony growth for yeast strains in the presence and absence (control) of sub-minimum inhibitory concentration of MMS (0.3%). The top 10% of the strains (31 in total) that was detected by GD as most sensitive to MMS was selected for further analysis. As expected from the molecular activity of MMS, this list contained a group of deletion strains for those genes that are functionally related to DDR such as; *pol32*, which encodes for a subunit of DNA polymerase delta required for error-prone DNA synthesis in the presence of DNA damage, *ubc30*, which encodes for a Ubiquitin-conjugating enzyme involved in the error-free DNA postreplication repair pathway, *rem50*, that we have recently shown to be involved in non-homologous end joining (NHEJ) pathway (unpublished data), etc. This further indicated that GD provides biologically meaningful data. Another group of genes that were identified by GD as part of the most sensitive strains were those that had no previous known functional relationship to DDR. These genes may represent a secondary target for MMS on the cell, or alternatively, they may represent novel genes that are involved in DDR. One such gene is *mek1*. It is previously reported that *mek1 *encodes for a protein kinase required for meiotic recombination [26]. But the involvement of *mek1 *in DDR has not been documented before. To examine a possible role for this gene in DDR, we subjected the yeast gene deletion strain for *mek1*, to a plasmid repair assay [27]. In this assay, yeast strains are transformed with equal amounts of intact and linearized (by restriction digestion) plasmids. The region around the site of linearization has no homology to the yeast chromosome. Therefore, the strains that are deficient in NHEJ pathway, which is responsible for repair of double stranded DNA breaks in the absence of a homologous DNA template, show reduced ability to circularize the plasmid and form fewer colonies on the selective media. It was observed that in the absence of *mek1*, yeast cells had approximately 80% relative reduction (Table 2) in plasmid repair. This suggests that *mek1 *can affect the efficiency of NHEJ in yeast cells. We note however, that further analysis is needed to investigate the molecular function of *mek1 *in NHEJ.

**Table 2:** Efficiency of yeast deletion mutants to repair linearized plasmid

Yeast strain	Colonies formed with intact plasmid	Colonies formed with linearized Plasmid	Relative repair efficiency
wild type	8429	8092	1.000
*yku70 *deletion	6953	587	0.088
*mek1 *deletion	7073	1241	0.183

*yku70 *is a major player in NHEJ pathway and its deletion strain is used as a control. Each experiment is repeated four times and the results are combined. The efficiency of the wild type to repair double stranded breaks is set at 1.000. The repair efficiencies for the other two strains are related to this value.

## Conclusion

Here we present a fast and efficient method termed GD analysis, to quantify yeast colony size. Using this approach, we show that the berberine is genetically linked to gene expression components, among other cellular processes. In addition, we show that *mek1 *is a novel gene associated with DNA damage in *S. cerevisiae*.

The work presented here offers significant improvement over the previously used method of manual inspection to estimate and compare yeast colony size under different experimental conditions. The speed and accuracy associated with this approach makes it an ideal choice for large-scale functional genomics investigations in which comparative colony size measurements are needed.

## Methods

### Colony Size Measurement

GD processes digital images taken from growth arrays of yeast colonies. The code for the GD software is written in MATLAB. Input images to the GD system for the experiments discussed in the present study are in Joint Photographic Experts Group (JPEG) format, but GD can process any image file format supported by MATLAB's image processing toolbox, such as TIFF (Tagged Image File Format), PNG (Portable Network Graphics), BMP (BitMap), and GIF (Graphics Interchange Format). All images are captured with a Hewlett-Packard PhotoSmart 735 camera. The width of each image is 2048 pixels and its height is 1536 pixels. Both horizontal and vertical resolutions are 72 dpi. The automated system performs three stages of preprocessing, processing and post-processing on the input images.

### Preprocessing

It is crucial to equalize the zoom, angle and size of input digital images. These parameters may vary among different images, however the system is designed to compensate for such differences in order to keep consistency among images and prevent error occurrence.

**Figure 2:**
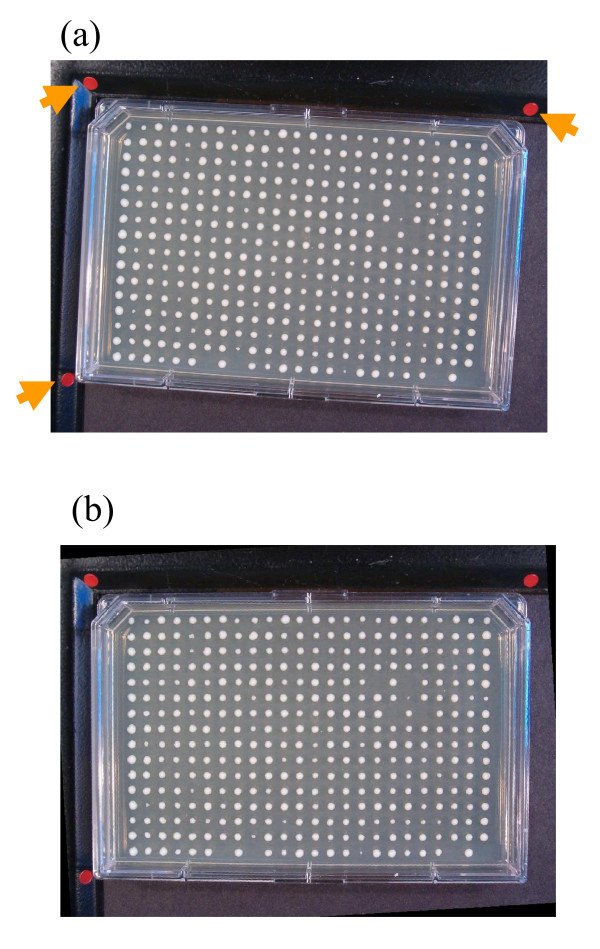
**Sample input image**. A sample input image to the system is shown in (a). Arrows show the red indicators. (b) Image in (a) is aligned using a linear conformal spatial transformation.

Through color recognition, the system detects three red stickers that indicate the plate's peripheral corners and consequently recognizes the approximate region occupied by the plate as shown in Figure 2(a). As is apparent in the example of Figure 2(a), the plate image may be tilted. The system aligns the image by taking pairs of control points and uses them to infer a spatial transformation. One set of control points, namely the input points, is the centroid coordinates of the top two red indicators. The other set is a pair of points on a precisely horizontal line, which are a fixed distance apart. The latter is called the alignment points and is the standard alignment we intend to achieve for the plate. The resizing scale and rotation angle needed to bring the image into the standard alignment are then calculated by the system. Figure 2(b) shows an image aligned using this method. Background objects such as plate frame, metallic tray, red indicators, plate name sticker, etc. are then cropped from the image, based on a fixed distance from the stickers.

GD is flexible to plate layouts. As long as the plate has a trapezoid form and the corner markers are present, plates with any dimensionality, rotation angle and colony arrangement can be accurately analyzed.

Image illumination is a key point for accurate image processing. Images resulting from poor (for example, non-uniform illumination) could be quite difficult to segment [28]. The possibility of nonuniform background illumination was investigated by subtracting the background image of each plate's image from the original image. It was observed that GD yielded results almost identical to when no illumination enhancement was performed. Examining the histogram of plate images, captured at different times, further indicated the consistency of illumination conditions between various images.

### Processing

**Figure 3:**
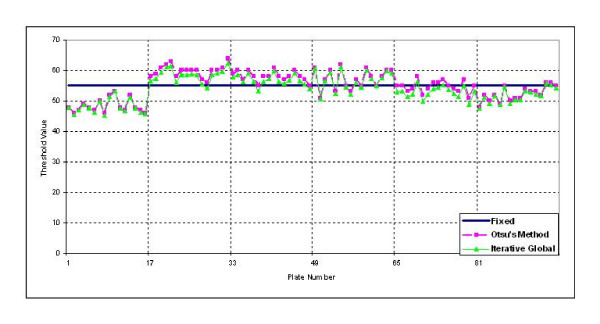
**Segmentation threshold value acquired by three different methods**. The values represent the data from 6 independent sets of plates. 96 plates of yeast arrays were used.

In order to extract the regions of interest (ROI), i.e., yeast colonies, we apply image segmentation. Three different thresholding techniques (fixed global, iterative global, and Otsu's method) were tested for this purpose. The fixed threshold was selected empirically. The iterative global method is a heuristic approach which seeks the valley of image histogram through an iterative process [28]. Otsu's method chooses the threshold to minimize the intra-class variance of the thresholded black and white pixels [29]. Figure 3 plots a comparison of these techniques for a total of 96 yeast colony plates (six independent sets and each set including sixteen plates). It can be seen from the plot, that the trend of iterative global thresholding and Otsu's method are very similar. It is also evident that they fluctuate close to the fixed threshold level. Further investigations revealed the superiority of Otsu's method over the iterative global thresholding technique. Comparison of segmentation results in random yeast array images shows an average 0.73% detection improvement when Otsu's method is used as the thresholding technique. Although this is a negligible difference, it is worth considering, because correct detection of every single pixel adds to the accuracy of the GD system. Hence, Otsu's technique was selected for segmenting the yeast array images. The result is a binary (black and white) image, in which any pixel with intensity greater than the threshold appears in white. That includes both the ROI and also those pixels which are not in fact ROI but exceed the intensity threshold value.

**Figure 4:**
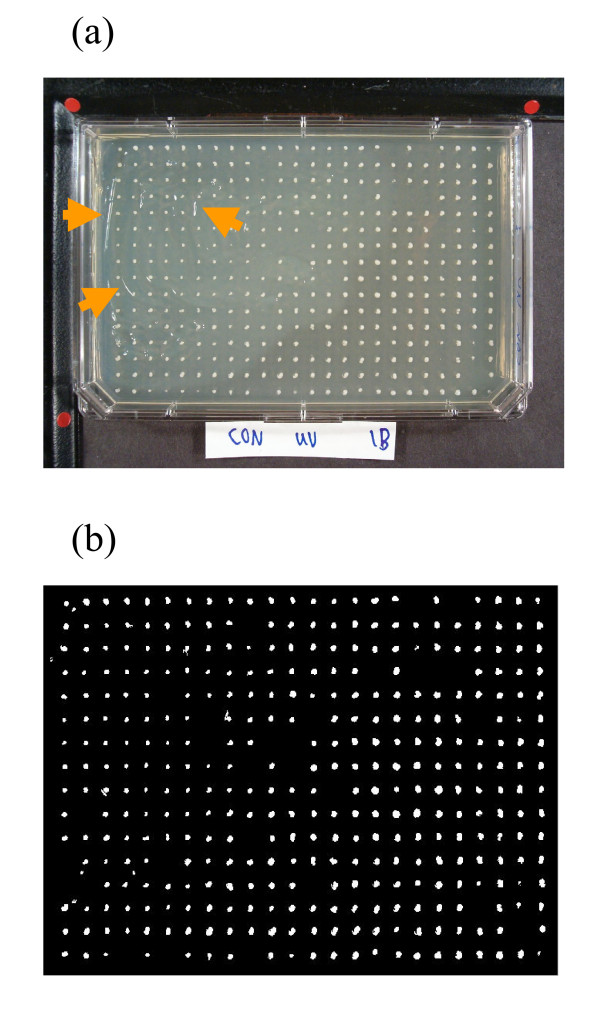
**The automated system can detect colonies from other undesired objects**. (a) Unwanted long entities and small blemishes are present in the image (arrows point to some instances). (b) The automated system correctly discards most of these objects as can be seen in the binary image after segmentation.

After segmentation, a size and shape analysis is performed to distinguish between true colonies and mistaken blemishes. Only those objects with size greater than 0.0005 of total area of bright pixels in thresholded image are reserved. The value 0.0005 is reasonably small so as to exclude undesired isolated pixels and has been selected experimentally. Also according to our *a priori *knowledge about the shape of regions of interest, we know that colonies normally keep a rather circular shape. Therefore by using morphological operations, the objects in the image are examined based on their eccentricity and consequently long entities are filtered. An example of segmented and filtered image is demonstrated in Figure 4. It should be noted that some mutants show no growth on a test condition. Such positions on the plate will be identified in the last phase of the processing stage, and they will be attributed a growth measure of zero in the final result of the system.

The next step of work is to provide a coordinate oriented map of colony areas. We designed and used an adaptive object specific mask to handle this issue. For every object (colony) present in the image, a mask is automatically formed, which is a rectangular area slightly bigger than the object. The dimensions of this mask are specific to the shape and size of that particular object. The sum of the bright pixels that fall inside the object specific mask is stored as the total area of that object in an array at the coordinate of the object's centroid. We have named this array "area map", as it in fact maps the total area of each object to its centroid. This method is advantageous in two ways. Firstly by applying the adaptive object specific mask, we ensure that an accurate measurement of colony area is performed (i.e. a part of the colony does not get eliminated or missed due to wrong window size). Secondly by sorting the area value of each colony at location of its centroid, we can keep track of the correlation between size and position of individual colonies later on.

The area map needs to be ordered according to a master gene list. Master gene list is a database containing thorough information about those genes that are being traced by the plate analysis research. According to the gene list, the bottom-right colony in each plate denotes colony 1, and the top-left colony in that plate represents colony 384 (for a 16 × 24 array). The objective of the ordering step is to come up with an array that has the results in order from coordinate (1, 1) to coordinate (16, 24). Very often the colonies do not appear in a strictly aligned grid. However since the system is aimed to work accurately without human interaction, such deviations need to be considered automatically. To solve this issue, we obtain the logical version of the array area map. The result, named "centroid map" is an array the same as the area map, having its non-zero elements replaced with 1 s. The peripheries of the map are calculated from prototype equations below:

*y_start *= *min_y_centroid *- round(*max_y_width*/2)

*x_start *= *min_x_centroid *- round(*max_x_width*/2)

*y_end *= *max_y_centroid *+ round(*max_y_width*/2)

**Figure 5:**
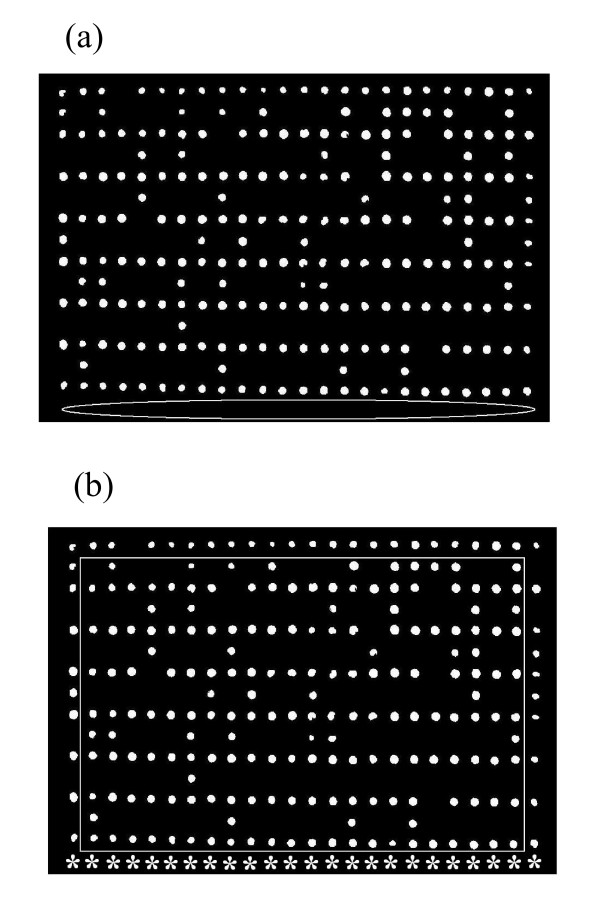
**Missing rows**. The bottom row of colonies is missing in (a). This is marked on the image by a white ellipse. (b) White rectangle showing position of distance checking limit. Each side of this rectangle is 100 pixels distant from the plate side parallel to it. Imposing the predefined periphery values (stars) compensates for the missing row.

*x_end *= *max_x_centroid *+ round(*max_x_width*/2)

where the first term at right hand side of equality is the minimum, as in equations (1) and (2), or maximum, as in equations (3) and (4), centroid coordinate along *x *or *y *direction. The second term at right side of equality is the maximum colony width value among all colonies in a plate image. Here occurrence of a worst case should be taken into account. There is a chance that from the four peripheral sequences of colonies, one whole row or column is missing. An example can be seen in Figure 5(a) where the bottom row of colonies does not exist. This leads to an incorrect calculation of *y_start *and *x_start *in equations (1) and (2). To compensate for such cases, a distance check has been designed to verify the coordinates of starting and ending margins. If any of the four values at left hand side of equations (1) to (4) is not less than a specific limit, then a predefined periphery value is imposed for that quantity. To illustrate this more clearly, in Figure 5(b), the rectangle is a visual representation of the mentioned limit. In the same figure, the imposed periphery value is shown as a sequence of stars imposed on the original image. As is evident, the star coordinates fall outside the limit rectangle, meaning calculation of *x_start *and *y_start *based on these points would now be correct. The limit is supposed to be 100 pixels distant from the image borderline for each side. The predefined periphery values are chosen experimentally based on the average location of colony centroids in many array images.

As the last step of the processing phase, the centroid map and area map are utilized to order the colonies. Beginning from *x_start *and *y_start *and heading for *x_end *and *y_end*, regions of *m n *size from centroid map are examined. For every nonzero value found in that region, its corresponding area value from area map is added to an accumulator for that region. Parameters *m *and *n *are attained from equations (5) and (6).

*m *= (*x_end*-*x_start*)/24

*n *= (*y_end*-*y_start*)/16

**Figure 6:**
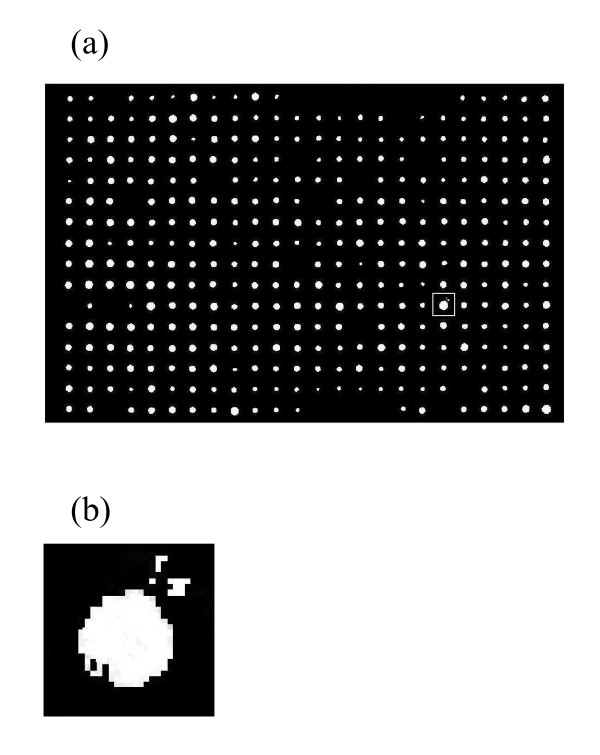
**Satellite effect around a colony**. (a) Example of a colony, which is comprised of a main region and a few smaller islands (satellites). (b) Zoomed in version of (a).

The role of accumulator is noteworthy because of heeding those objects (colonies), which have small disconnected parts. Although the colonies are usually compact objects with one definite centroid, there are cases where a colony is comprised of a main region with a few smaller islands around it (see an example in Figure 6). Without the accumulator, the ordering algorithm would only consider the area of the first object detected in the examination region as the colony area in that coordinate. With the accumulator however, the system continues the process of centroid detection and adding to the accumulator until there is no more nonzero centroid left in the examination region. At positions where no colony growth has occurred, no nonzero value is found inside the *m *× *n *window surrounding that position, and hence a total area of zero is recorded for that position.

A check is executed when the ordering step is finished. This check compares the total value of white pixels before and after ordering. If these values are not identical, it is revealed that some information has been missed in the midst of ordering.

### Post processing

Some statistical analysis is performed on the output of the processing stage. For every plate the average value of white pixels is calculated from equation (7).

Save=1/N∑i=1NSi
 MathType@MTEF@5@5@+=feaafiart1ev1aaatCvAUfKttLearuWrP9MDH5MBPbIqV92AaeXatLxBI9gBaebbnrfifHhDYfgasaacH8akY=wiFfYdH8Gipec8Eeeu0xXdbba9frFj0=OqFfea0dXdd9vqai=hGuQ8kuc9pgc9s8qqaq=dirpe0xb9q8qiLsFr0=vr0=vr0dc8meaabaqaciaacaGaaeqabaqabeGadaaakeaacqWGtbWudaWgaaWcbaGaemyyaeMaemODayNaemyzaugabeaakiabg2da9iabigdaXiabc+caViabd6eaonaaqadabaGaem4uam1aaSbaaSqaaiabdMgaPbqabaaabaGaemyAaKMaeyypa0JaeGymaedabaGaemOta4eaniabggHiLdaaaa@3F48@

where *N *is the total number of white objects present in plate and *S*_*i *_is the area of object *i*.

The deviation of area of each object from plate's average area is calculated by subtracting the scalar *S*_*ave *_from the plate's 1D ordered area array explained in equation (8).

*ΔS*_*i *_= *S*_*i *_- *S*_*ave*_; *i *= 1, ..., 384 (16 × 24 = 384)

The reader is reminded that the ultimate objective of the discussed computerized analysis is to monitor the colony area change in two corresponding experiments, in one of which the yeast colonies are treated with a specific kind of chemical/drug and in the other one, they are left untreated (control). Thus, the area difference and percentage of reduction between the two plates plus the normalized value of these quantities are also calculated. That provides a thorough understanding of area change for each colony within its plate and also compared with its corresponding colony in another plate.

### Yeast manipulations

The set of non-essential yeast gene deletion strains has been described elsewhere [30]. The array of gene deletion strains was replicated by pinning method using a 384-floating pin replicator (VP384F). Unless stated otherwise, the yeast cells were grown on YPD media using Omni plates (Nunc Inc., Rochester, NY, USA) and incubated for 1–2 days. Images taken from the plates were used for further analysis. Colonies that appeared irregular were eliminated from the data. The pinning experiment with cobalt was repeated three times. Growth differences that were confirmed at least once were reported. For strain sensitivity, colony growth reduction of 30% or more was considered as significant and was used for sensitivity assignment. Based on the function of their gene deletion, the sensitive strains were clustered in groups, which at least contained three strains. For the plasmid repair assay, a derivative of p416 plasmid [31] was used. The *XbaI *site was used to linearize the plasmid.

## Availability and requirements

Project name: Growth Detector (GD)

Operating system: Windows XP

Programming language: MATLAB

Licence: For scientists wishing to use the GD software for non-commercial purposes, the software is available free of charge from the authors and without the need for a software transfer agreement. For commercial users a licence is required.

## Authors' contributions

AG, JA, MS, JX and MZ developed the project, all authors participated in its design, NM designed and implemented the GD software under supervision of JA, MJ and AG provided the DNA repair data, NMR and MS provided the berberine extract data, MJ and AG provided the cobalt sensitivity data, and AG, NM, MJ and NMR drafted the manuscript. All authors read and approved the final manuscript.
